# Pulchinenoside C Attenuates the Development of Osteoarthritis by Inhibiting the PI3K/AKT/NF‐κB Signalling Pathway

**DOI:** 10.1111/jcmm.70738

**Published:** 2025-08-01

**Authors:** Jiawei Hu, Kai Xiao, Jianhui Liang, Xiaolong Yu, Meisong Zhu, Zhihui Kuang, Shoujie Shi, Bin Zhang, Qiang Xu

**Affiliations:** ^1^ Department of Orthopedics The 1st Affiliated Hospital, Jiangxi Medical College, Nanchang University Nanchang Jiangxi China; ^2^ Department of Orthopedics Nanchang Hongdu Traditional Chinese Medicine Hospital Nanchang Jiangxi China; ^3^ Postdoctoral Innovation Practice Base The First Affiliated Hospital, Jiangxi Medical College, Nanchang University Nanchang China

**Keywords:** cartilage degeneration, osteoarthritis, PI3K/AKT/NF‐κB, pulchinenoside C, pyroptosis

## Abstract

Osteoarthritis (OA), the most prevalent type of arthritis, is characterised by permanent damage to the articular cartilage. The progression of OA is mediated by the disruption of extracellular matrix (ECM) homeostasis and the overactivation of the inflammatory response. Herbal extracts, with their safety and multi‐targeting properties, have demonstrated unique advantages in inhibiting inflammation, delaying cartilage degeneration and regulating the joint microenvironment. The PI3K/AKT signalling and NF‐kB signalling pathways, two classical inflammatory signalling pathways, mediate the occurrence and development of osteoarthritis by regulating the inflammatory response. Pulchinenoside C (PC, also known as Anemoside B4), derived from Pulsatilla chinensis, contains the highest concentration of triterpenoid saponins. PC exerts definite anti‐inflammatory effects. However, its ability to delay the progress of OA by regulating the classical inflammatory signalling pathway remains to be clarified. This study aimed to elucidate the mechanism through which PC prevented the progression of OA in vitro and in vivo. In vitro, PC exerted significant anti‐inflammatory effects on IL‐1β‐induced inflammatory responses in the ATDC5 cells. Notably, PC also inhibited matrix metalloproteinase expression and successfully protected the extracellular matrix of chondrocytes. In vivo, PC prevented the development of OA in a C57BL/6 mouse model of OA caused by medial meniscus (DMM) instability by inhibiting the activation of the PI3K/AKT/NF‐κB pathway. These findings suggest that PC is a potentially safe and successful therapeutic agent for OA.

## 1 Introduction

Osteoarthritis (OA) is characterised by damage to the articular cartilage, osteophyte formation, changes in the subchondral bone, varying degrees of synovitis and thickening of the joint capsule [1]. Age‐related increases in the prevalence of OA have led to it becoming a widespread joint illness worldwide [2]. Pain management and total knee replacement, the currently available treatment strategies for OA, yield subpar functional results and have a negative impact on quality of life [3]. The precise pathophysiology of OA remains unknown; however, inflammation plays a critical role in the development of OA [4]. Anti‐inflammatory treatment targeting inflammatory factors has demonstrated beneficial effects in patients with OA [5, 6]. The activation of the nuclear factor‐κB (NF‐κB) pathway plays a vital role in activating the inflammatory response during OA [7]. Activated NF‐κB is transferred to the nucleus from the cytoplasm in response to the release of inflammatory mediators such as interleukin‐1 (IL‐1β). This activates inflammation‐related response genes such as matrix metalloproteinases (MMPs) and a disintegrin and metalloproteinase with thrombospondin motifs (ADAMTS) [8]. The overexpression of MMPs and ADAMTS, which break down type II collagen (COL2a1) and aggrecan (a significant structural proteoglycan in cartilage) and induce chondrocyte apoptosis, aggravates the development of OA [9, 10]. Thus, formulating effective and safe strategies to delay the progression of OA is imperative.

Inflammation, an innate immune process that occurs in response to physical, physiological and/or oxidative stress in the body, plays a central role in the development of OA [11]. The PI3K/AKT signalling pathway activates the NF‐κB signalling pathway and promotes its translocation into the nucleus through phosphorylation [12, 13]. Furthermore, the PI3K/AKT/NF‐κB signalling cascade induces inflammation in chondrocytes, leading to an increase in the expression of MMPs, deintegrins and ADAMTS, and a decrease in the expression of type II collagen and aggrecan [14, 15]. Inhibition of the PI3K/AKT signalling pathway can reduce the inflammatory response associated with OA [1, 16]. Thus, medications targeting the PI3K/AKT/NF‐κB signalling pathway represent a trustworthy and efficient modality for the treatment of OA.

The use of herbal extracts for the management of OA has become increasingly popular in recent years [17]. Triterpenoid saponin, found in *Pulsatilla chinensis*, pulchinenoside C (PC, also known as anemoside B4), has demonstrated definite anti‐inflammatory activity [18]. PC prevents the activation of the PI3K/AKT signalling pathway by hepatocellular cancer cells [19]. Furthermore, it exerts anti‐inflammatory effects by inhibiting the NF‐κB signalling pathway [20, 21].

PC may serve as a novel therapeutic agent for the management of OA by inhibiting the PI3K/AKT/NF‐κB signalling pathway. This study aimed to evaluate the anti‐inflammatory effect of PC on IL‐1β‐induced OA in vitro and the mechanism through which it inhibits the occurrence and development of early arthritis in mice.

## 2 Materials and Methods

### 2.1 Chemicals, Media and Reagents

**FIGURE 1:**
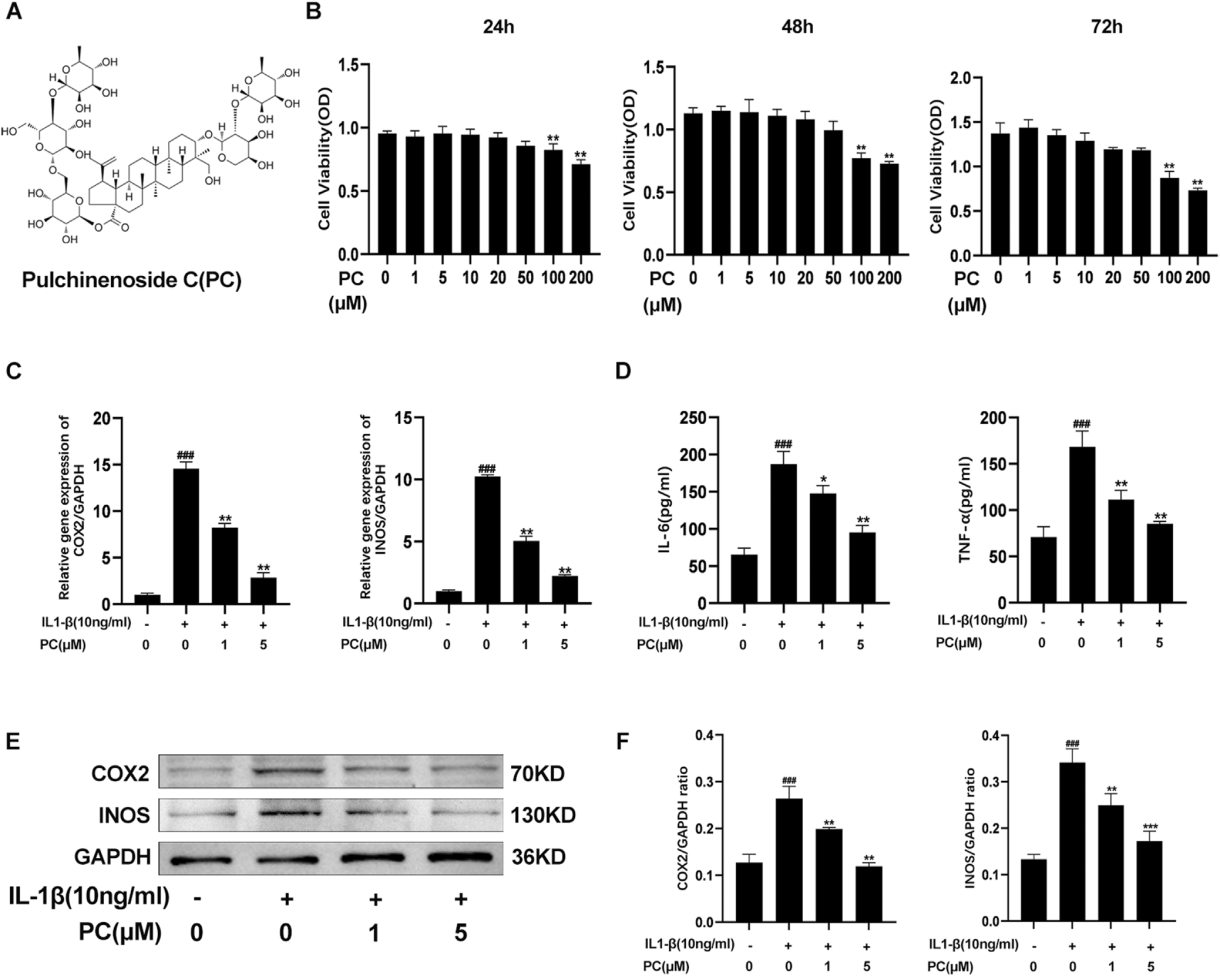
Effect of PC on the activity of chondrocytes and its inflammatory inhibition on chondrocytes. (A) The chemical structure of PC. (B) Effects of PC on the ADTC5 cells were determined in response to increasing concentrations for 24, 48 and 72 h. (C) The expression levels of iNOS and COX‐2 mRNA were measured using qRT‐PCR. (D) The expression of IL‐6 and TNF‐α in the ADTC5 cell supernatants was detected using ELISA. (E) The expression levels of iNOS and COX2 after treatment with different concentrations of PC were detected using western blotting and quantification analysis in (F). The values presented are the mean ± SD of three independent experiments. **p < 0.01, compared with the control group in Figure (B). ^###^ *p* < 0.001 versus the control group and *p < 0.05, **p < 0.01, ***p < 0.001 versus the IL‐1β alone treatment group.

Figure 1A presents the molecular structure of PC (purity ≥ 98.0%) purchased from MedChem Express. A stock solution (10 mM) of PC, obtained by dissolving it in dimethyl sulfoxide, was stored at −80°C in the dark. Betulinic acid (BetA), 740Y‐P and celecoxib were purchased from MCE. DMEM/F12 medium, purchased from KeyGEN BioTECH (Suzhou, China), was supplemented with 80 U/mL of penicillin and 0.08 mg/mL of streptomycin. Foetal bovine serum (FBS), cell counting kit‐8 (CCK‐8), toluidine blue solution, fluorescent secondary antibodies and 4′,6‐Diamidino‐2‐phenylindole (DAPI) were purchased from Gibco (Grand Island, New York, USA), APEXBIO (Houston, USA), Solarbio (Beijing, China), Elabscience (Wuhan, China) and Solarbio (Beijing, China), respectively.

### 2.2 Cell and Cell Culture

Chondrogenic ATDC5 cell line, purchased from Riken Cell Bank (Ibaraki, Japan), was cultured in DMEM/F12 medium supplemented with FBS (5%). ATDC5 cell line was cultured in a medium supplemented with ITS (10 μg/mL of insulin, 5.5 μg/mL of transferrin and 6.7 ng/mL of sodium selenite; Invitgen) for 2 weeks to induce chondrocyte differentiation of the ADTC5 cells [22]. The cells were cultured in an incubator with 5% CO2 at 37°C.

### 2.3 Cell Viability Assay

The effect of PC on the survival rate of ATDC5 cells was assessed using a CCK‐8 assay kit. In brief, ATDC5 cells were inoculated into 96‐well plates at a density of 5 × 10^**3**^ cells/well, with 4 wells/test concentration. The cells were treated with different concentrations of PC (0–200 μmol) for 24, 48 and 72 h after they had adhered completely to the wells. Then, an appropriate amount of CCK‐8 solution was added to each well to 10% of the total liquid volume of each well. The cells were cultured in a humidified atmosphere with 5% CO_2_ for 1–2 h at 37°C, and the absorbance at 450 nm was measured using a multifunctional microplate reader (Thermo Scientific, Varioskan lux, Shanghai, China).

### 2.4 High‐Density Culture and Toluidine Blue Staining

The ATDC5 cells were cultured using a high‐density culture method [23]. In brief, 10 μL of the ATDC5 cell suspension was inoculated in 24‐well plates at a density of 2 × 10^7^ cells/well in triplicate. The inoculated ATDC5 cells were placed in an incubator, and cell adhesion was observed every 20 min. Following the complete adhesion of the cells to the wells, 500 μL of DMEM/F12 supplemented with FBS (5%) and penicillin/streptomycin (1%) was added to each well, and the cells were cultured for 24 h. IL‐1 β (10 ng/mL) and different concentrations of PC (0, 5, 10 μM) were added to the cells thereafter, and the medium was changed every 2 days. The medium was removed after 7–9 days of culture, and the cells were rinsed thrice with phosphate‐buffered saline (PBS), fixed with 4% paraformaldehyde for 30 min, and stained with toluidine blue. ImageJ software (National Institutes of Health, Bethesda, Maryland, USA) was used to calculate the average optical density to determine the staining intensity.

### 2.5 RNA Extraction and Quantitative Real‐Time PCR (qRT–PCR)

The ATDC5 cells were seeded in 6‐well plates at a density of 2 × 10^5^ cells/well and stimulated with IL‐1β (10 ng/mL) with or without PC (1.5 μM) for 48 h. TRIzol reagent (Thermo Fisher Scientific, USA) was used to extract total RNA from the cells, and RNA (1 μg) was reverse transcribed to complementary DNA using reverse transcriptase (TaKaRa, Japan) in accordance with the manufacturer's instructions. The purity and quality of cDNA were verified by calculating the A260/A280 ratio. RT‐PCR was performed using TB Green Premix Ex Taq II (TaKaRa) with CFX Connect (Bio‐Rad, Shanghai, China) as follows: denaturation at 95°C for 5 s and annealing at 60°C for 40 s for 40 cycles. The 2^−ΔΔCt^ method was used to analyse gene expression with glyceraldehyde‐3‐phosphate dehydrogenase (GAPDH) levels as the internal control. The primer sequences used were as follows: GAPDH forward (5′‐TCTCCTCTGACTTCAACAGCGAC‐3′) and reverse (5′‐CCCTGTTGCTGTAGCCAAATTC‐3′); COL2a1 forward (5′‐CTCAAGTCGCTGAACAACCA‐3′) and reverse (5′‐GTCTCCGCTCTTCCACTCTG‐3′); SOX9 forward (5′‐GCAGGCGGAGGCAGAGGAG‐3′) and reverse (5′‐GGAGGAGGAGTGTGGCGAGTC‐3′); iNOS forward (5′‐CTCTTCGACGACCCAGAA AAC‐3′) and reverse (5′‐CAAGGCCATGAAGTGAGGCTT‐3′); COX‐2 forward (5′‐CACCCTGACATAGACAGTGAAAG‐3′) and reverse (5′‐CTGGGTCACGTTGGATGAGG‐3′); MMP13 forward (5′‐TGTTTGCAGAGCACTACTTGAA‐3′) and reverse (5′‐CAGTCACCTCTAAGCCAAAGAAA‐3′); and ADAMTS‐5 forward (5′‐GCAGAACATCGACCAACTCTACTC‐3′) and reverse (5′‐CCAGCAATGCCCACCGAAC‐3′). Each gene was analysed at least thrice.

### 2.6 Enzyme‐Linked Immunosorbent Assay

The ADTC5 cells were treated with IL‐1β or PC for 72 h in accordance with the manufacturer's instructions, and the supernatant was collected to measure the pro‐inflammatory cytokine levels. In brief, enzyme‐linked immunosorbent assay (ELISA) kits were used to measure the TNF‐α and IL‐6 levels. Horseradish peroxidase (HRP)‐coupled affinity proteins were added to each microplate well and incubated. TMB substrate solution was added, and the enzyme‐substrate reaction was terminated through the addition of sulphuric acid solution. A spectrophotometer was used to detect the changes in the colour at a wavelength of 450 ± 10 nm. The OD values of the samples were compared with a standard curve to determine the expression levels of pro‐inflammatory cytokines in each sample.

### 2.7 Western Blot Analysis

The ADTC5 cells were inoculated on 6‐well plates and subjected to two different treatments. The cells were stimulated with IL1‐β (10 ng/mL) and different concentrations of PC (1 or 5 μM) for 2 days. Pathway proteins were extracted after 6–8 h of starvation in serum‐free DMEM/F12 medium supplemented with PC (1 or 5 μM) in advance and 20 min of treatment with IL1‐β (10 ng/mL). A radioimmunoprecipitation assay (RIPA) buffer containing protease and phosphate inhibitors (MedChemExpress, Monmouth, NJ, USA) was used to extract the total proteins harvested from the treated ATDC5 cells. The lysates were placed on ice for 30 min, mixed every 5 min on a vortex shaker and centrifuged at 12,000 rpm for 15 min at 4°C. The protein concentration in the supernatant was determined using the BCA protein detection kit in accordance with the manufacturer's instructions. Sodium dodecyl sulphate‐polyacrylamide gel electrophoresis was performed to separate the protein samples (30 μg/well), which were transferred onto polyvinylidene fluoride membranes. The membranes were blocked with 5% nonfat milk overnight at 4°C, followed by incubation with primary antibodies for at least 8 h at 4°C. The membranes were rinsed thrice with TBST and incubated with secondary antibodies (1:5000) for 2 h at 24°C. UVP Chemstudio touch (Analytik Jena AG, Germany) was used to detect the bands on the membranes. ImageJ software was used to quantify the strength of the blots, with GAPDH as the internal control.

### 2.8 Nucleus Protein Extraction

The ADTC5 cells were inoculated on 6‐well plates. The treated cells were rinsed with PBS and collected using a cell scraper. The supernatant was removed after centrifugation. The mixture was swirled at high speed for 15 s following the addition of 250 μL of cytoplasmic protein extraction reagent and placed on ice for 10 min. Cytoplasmic protein was obtained as the supernatant after centrifuging the mixture at high speed for 10 min. A nuclear protein extraction reagent (100 μL) was added and mixed for 15 s. Nuclear protein was obtained as the supernatant after centrifuging the mixture at high speed for 10 min. The nucleoprotein extraction kit was purchased from Solarbio.

### 2.9 Immunofluorescence Staining

The ADTC5 cells were inoculated on a 24‐well plate and cultured to approximately 50% confluence. The cells were starved for 1–4 h in serum‐free DMEM/F12 medium and treated with IL‐1β (10 ng/mL) for 30 min. The cells were fixed with 4% paraformaldehyde for 20 min, permeabilised with 0.5% Triton X‐100 for 15 min and blocked with bovine serum albumin for 30 min at room temperature. The cells were incubated overnight with anti‐p65 antibodies (1:500) at 4°C, followed by the corresponding fluorescent secondary antibodies at room temperature for 2 h. The cells were treated in the dark following the addition of the fluorescent secondary antibodies. The nuclei were stained with DAPI for 10 min in a humid environment, and observed under an inverted fluorescence microscope (Axio Observer+Axiocam 208, Zeiss).

### 2.10 DMM‐Induced OA Mouse Model

All animal experimental procedures were approved by the Animal Ethics Committee of Nanchang University (DM20210410). Destabilisation of the medial meniscus (DMM) surgical instability model was selected to mimic OA (OA) as DMM‐induced OA is similar to spontaneous OA lesions observed in older mice [24]. In brief, 24 C57BL/6 mice (male, 12 weeks of age) were anaesthetised through the intraperitoneal injection of 0.5% pentobarbital. The medial meniscal ligament was exposed through blunt dissection of the adipose and synovial tissues, and the medial meniscal ligament was transected with a scalpel. The animals were divided into four groups at random: non‐DMM control (sham), DMM p, DMM with 10 mg/kg celecoxib and DMM with 10 mg/kg PC groups. The mice included in the sham operation group underwent right knee arthrotomy without medial meniscal ligament resection. Disinfection with iodine solution was performed after all surgical procedures to prevent postoperative infections. The mice in the PC treatment group received intraperitoneal injections of 10 mg/kg PC on alternate days for eight consecutive weeks. The mice in the positive control (celecoxib) group received 10 mg/kg celecoxib administered using the same administration protocol. The mice in the sham operation and DMM model groups received equivalent volumes of PBS. The animals were maintained under standard housing conditions with ad libitum access to food and water throughout the study period. All mice were euthanised at the 8‐week postoperative endpoint, and the knee joint tissue was harvested for histological evaluation.

### 2.11 Micro‐Computed Tomography

The knee joint tissue samples acquired from the C57BL/6 mice were subjected to micro‐computed tomography (micro‐CT) analysis using a Micro‐CT 50 system (Scanco Medical, Switzerland). The scanning parameters were as follows: voxel size, 10 μm resolution; voltage, 100 kV; and current, 98 μA. Three‐dimensional (3D) reconstruction images were obtained and the related parameters [bone volume/tissue volume fraction (BV/TV), trabecular thickness (Tb.Th) and trabecular number (Tb.N)] were analysed using the micro‐CT system.

### 2.12 Histological Assessment

The knee joints of the mice in each group were fixed in 4% paraformaldehyde for 24 h and decalcified with 10% EDTA for 2 weeks. The joints were embedded in paraffin and sectioned to a thickness of 5 μm in the sagittal plane. The sections were stained with haematoxylin–eosin (H&E) and safranin O‐Fast Green. In addition, histological sections were stained with immunofluorescence and immunohistochemistry. The damage to the articular cartilage was categorised into eight grades (0 corresponding to normal, and 6 corresponding to vertical clefts/erosion to the calculated cartilage extension > 75% of the artistic surface) in accordance with the International Association for the Study of OA (OARSI) scoring system [25]. Five typical fields of view were selected at random from each slice and analysed under an optical microscope (Nikon, Tokyo, Japan).

### 2.13 Molecular Dynamics

PDB format files of AKT and p65 receptors were downloaded from the PDB database (https://www.rcsb.org/), and the 3D conformation format files of PC from the PubChem database (https://pubchem.ncbi.nlm.nih.gov/). AKT1 (PDB ID: 1UNQ) and P65 (PDB ID: 2O61) were selected for molecular dynamics (MD) targeting with PC. The Amber24 molecular dynamics simulation platform was used in this study. The protein system was parameterised using the ff19SB force field, and the solvent environment was selected as a high‐precision OPC water model. The constructed periodic water box ensured that the system was completely solventised. The cut‐off value of electrostatic interactions and van der Waals interactions was set as 1.0 nm, the time step was set as 2 fs and the long‐range electrostatic interactions were corrected using the PME algorithm to ensure accurate handling of the non‐bonding interactions. The system was constructed under constant temperature and pressure (300 K, 1 bar) for one composite system. Energy minimisation was performed after building the system, followed by NVE equilibrium kinetics at 200 ps and NPT equilibrium kinetics at 100 ps. The V‐rescale method was used to maintain the temperature of the hot‐bath coupled system, and the Parrinello‐Rahman method was used for pressure control. Lastly, 100 ns MD sampling was performed. The root mean square deviation (RMSD), root mean square fluctuation (RMSF), radius of gyration (Rg) and dynamic changes in the hydrogen bonding network were calculated using Amber's own module, Cpptraj and Python scripts to comprehensively assess the conformational stability and interaction characteristics of the complex system.

### 2.14 AKT1 Overexpression

The AKT1 overexpression plasmid was created by inserting the coding sequence (CDS) of NM_009652.4 into the pEX‐3 (pGCMV/MCS/Neo) vector under the control of the CMV promoter. The ADTC5 cells were transfected with the recombinant plasmid using Lipofectamine 3000 transfection reagent in accordance with the manufacturer's instructions, and incubated in complete medium for 48 h to enable AKT1 overexpression prior to downstream functional analyses.

### 2.15 Statistical Analysis

Data are presented as the mean ± SD (standard deviation). All experiments were performed independently in triplicate. One‐way analysis of variance (ANOVA) was performed with SPSS V.23.0 (SPSS Inc., Chicago, USA) to assess inter‐group differences. The Kruskal–Wallis H test was used to analyse non‐parametric data. A *p*‐value of < 0.05 was considered statistically significant.

## 3 Results

### 3.1 Effects of PC on ADTC5 Cell Viability

The CCK‐8 assay was performed to evaluate the cytotoxic effects of PC on the ADTC5 cells. The ATDC5 cells were incubated with various concentrations of PC (0, 1, 5, 10, 20, 50, 100, or 200 μM) for 24, 48 and 72 h after being seeded into 96‐well plates at a density of 5 × 10^3^ cells/well. Each experimental concentration was repeated in 3 wells. The CCK‐8 assay revealed that 200 μM of PC had an impact on cell viability after 24 h, and that effect was more obvious after 72 and 48 h when the concentrations were 100 μM and 200 μM (Figure 1B). Follow‐up experiments were performed with 1 μM and 5 μM PC, with 1 μM PC being the low‐dose treatment group and 5 μΜ PC being the high‐dose treatment group.

### 3.2 Effect of PC on the Expression of iNOS, COX2, TNF‐α and IL‐6 in IL‐1β‐Induced ADTC5 Cells

RT‐qPCR was performed to determine the expression of iNOS and COX‐2 in the ADTC5 cells following treatment with different concentrations of PC (1 μM and 5 μM) and IL‐1 (10 ng/mL) to explore the role of PC in IL‐1β‐induced inflammation. Treatment with IL‐1β resulted in a significant increase in the expression of the inflammatory factors iNOS and COX‐2 compared with that in the control group (Figure 1C). In contrast, treatment with PC resulted in a significant reduction in the IL‐1β‐induced up‐regulation of iNOS and COX2 expression at the mRNA level; this effect was dose‐related (1 μM and 5 μM). Treatment with PC resulted in a reduction in IL‐1β‐induced TNF‐α and IL‐6 expression (Figure 1D). Consistent with the results of RT‐qPCR, treatment with PC downregulated the protein expression of iNOS and COX‐2 in a dose‐dependent manner (Figure 1E,F). These findings demonstrate that PC suppressed the release of inflammatory mediators in a concentration‐dependent manner at the mRNA and protein levels.

### 3.3 PC Protects Against IL1‐β‐Induced Degradation of the Extracellular Matrix in ATDC5 Cells

**FIGURE 2:**
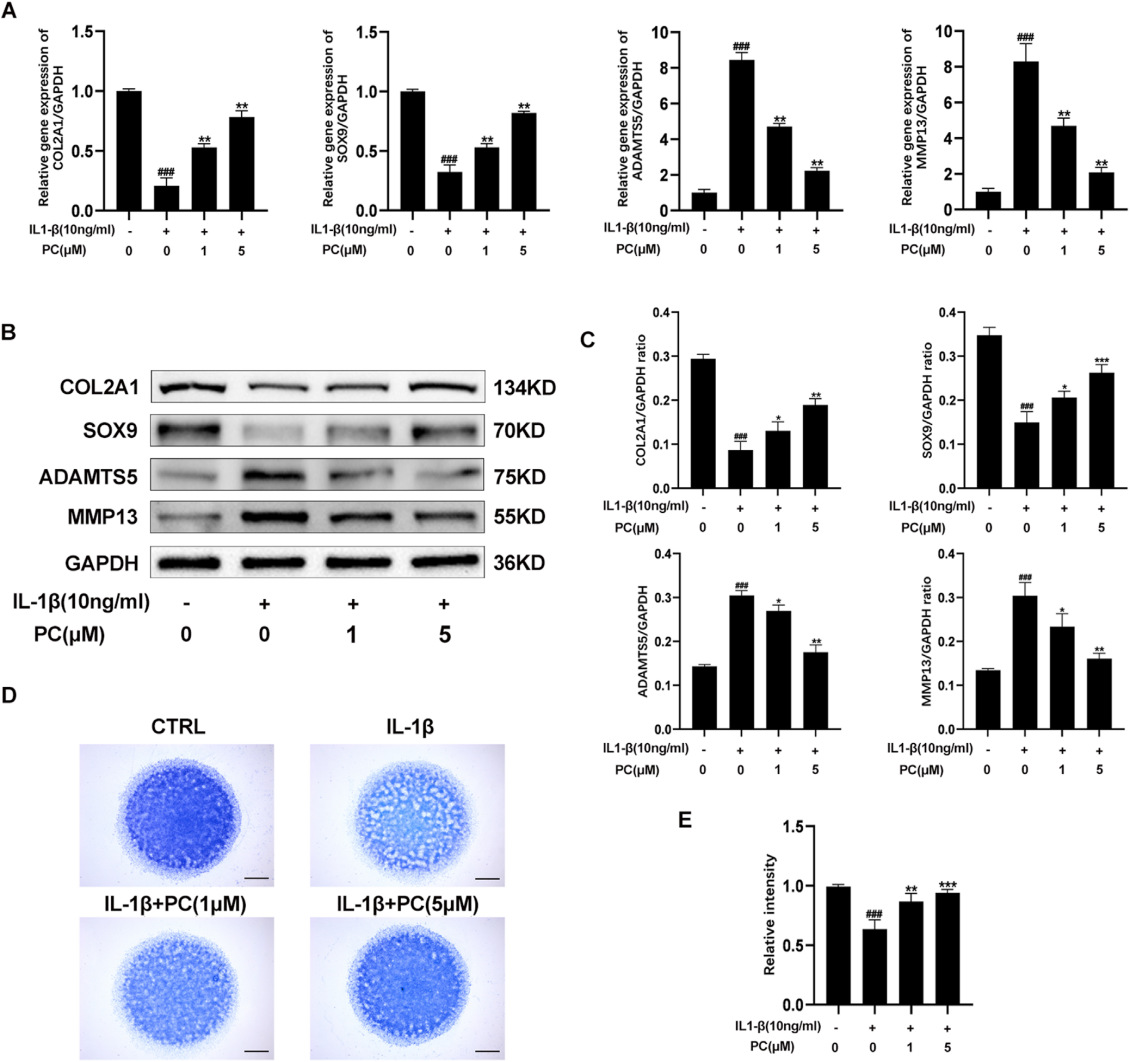
Protective effects of PC on the chondrocyte extracellular matrix. (A) The expression levels of COL2a1, SOX9, ADAMTS and MMP13 mRNA were measured using qRT–PCR. (B, C) The expression levels of COL2a1, SOX9, ADAMTS and MMP13 after treatment with different concentrations of PC were detected using western blotting and quantification analysis. (D) Toluidine blue staining results for ADTC5 cells cultured with IL‐1β (10 ng/mL) and various concentrations of PC (1 and 5 μM) for 7 days by high‐density culture. Scale bar = 200 μm (E) The relative intensity of toluidine blue‐positive cells. The values presented are the mean ± SD of three independent experiments. ^###^ *p* < 0.001 versus the control group and *p < 0.05, **p < 0.01 and ***p < 0.001 versus the IL‐1β alone treatment group.

**FIGURE 3:**
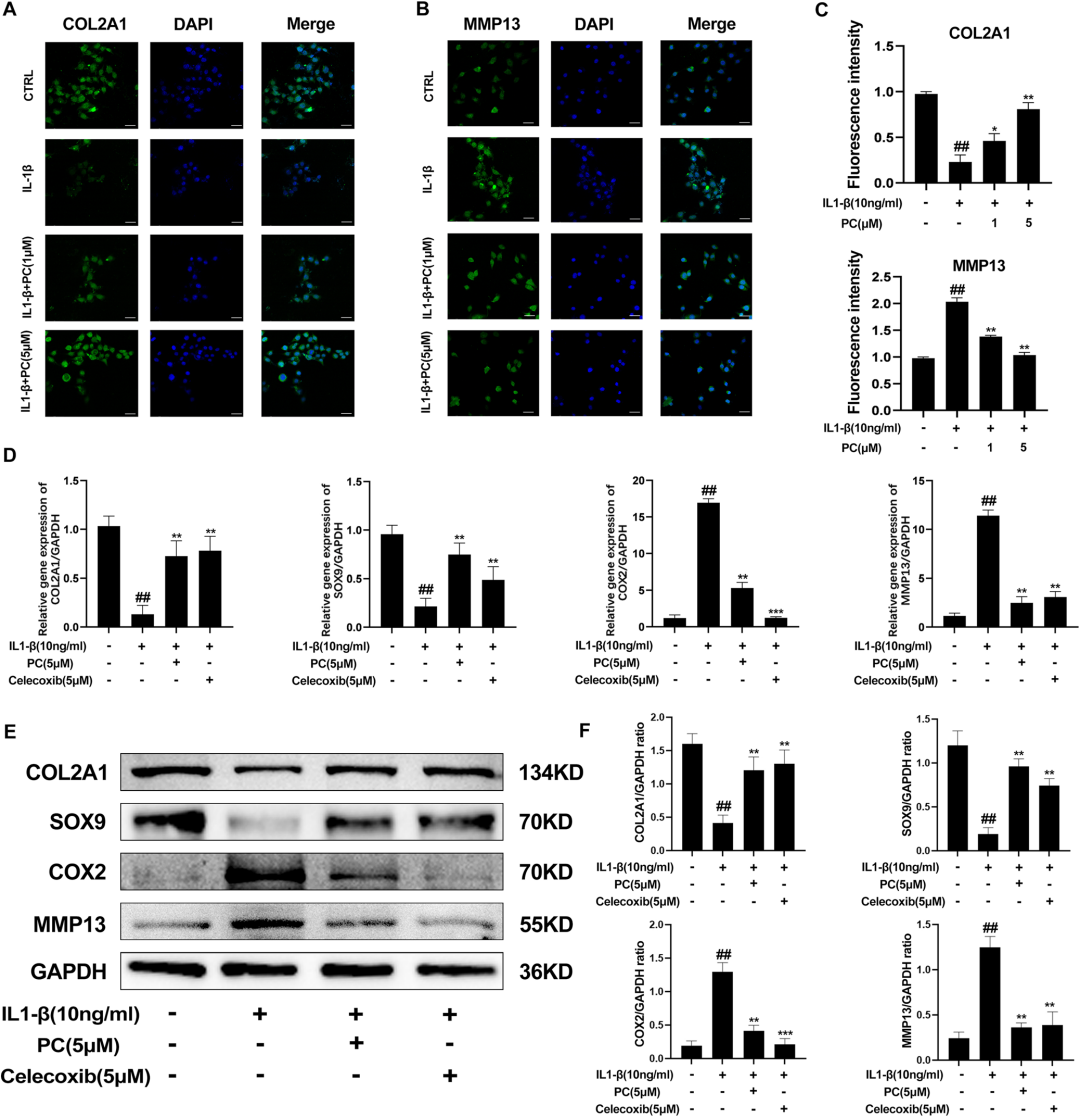
PC protects the cartilage extracellular matrix by inhibiting the NF‐κB signalling pathway. (A) Typical collagen‐II was detected by immunofluorescence combined with nuclear DAPI staining. Scale bar = 50 μm (B) MMP13 was detected by immunofluorescence combined with nuclear DAPI staining. Scale bar = 50 μm (C) Fluorescence intensity of COL2a1 and MMP13. (D) The mRNA expression levels of COL2a1, SOX9, COX‐2 and MMP13 were detected using qRT‐PCR in cells treated with PC or the positive control drug celecoxib. (E, F) The expression levels of COL2a1, SOX9, COX‐2 and MMP13 after treatment with PC or celecoxib were detected using western blotting and quantification analysis. The values presented are the mean ± SD of three independent experiments. ##*p* < 0.01 versus the control group and *p < 0.05, **p < 0.01, ***p < 0.001 versus the IL‐1β alone treatment group.

The effects of PC on the extracellular matrix (ECM) of IL‐1β‐induced cartilage precursor ADTC5 cells were assessed. IL‐1β stimulation resulted in a dramatic increase in the MMP‐13 and ADAMTS‐5 mRNA levels; however, the Col2a1 and SOX9 mRNA levels were significantly reduced. Treatment with PC resulted in the downregulation of MMP‐13 and ADAMTS‐5 and the upregulation of Col2a1 and SOX9 in a dose‐dependent manner (Figure 2A). The RT‐qPCR and Western blot results were consistent (Figure 2B,C). Toluidine blue staining of the high‐density cultures revealed that treatment with PC prevented the degeneration of the ECM in a dose‐dependent manner (Figure 2D,E). Immunofluorescence staining revealed that treatment with PC slowed the degradation of COL2a1 while inhibiting the expression of MMP13 in the ADTC5 cells (Figure 3A,B). Fluorescence quantitative analysis also supported this result (Figure 3C). These findings indicate that PC impairs the degradation of ECM in OA chondrocytes and has a significant protective effect on chondrocytes. Comparison between the therapeutic effects of PC and Celecoxib, a commonly used clinical drug for OA, using RT‐qPCR revealed that PC exhibited effects comparable with those of Celecoxib in inhibiting IL‐1β‐induced MMP‐13 expression and COL2A1 degradation. PC demonstrated superior efficacy in the prevention of SOX9 degradation, whereas Celecoxib was particularly effective in inhibiting COX2 expression (Figure 3D). Western blot analysis confirmed these findings (Figure 3E,F). These findings indicate that PC specifically inhibits inflammatory factors and OA in vitro.

### 3.4 PC Inhibits IL1‐β‐Induced Activation of the NF‐κ B Signalling Pathway in ADTC5 Cells

**FIGURE 4:**
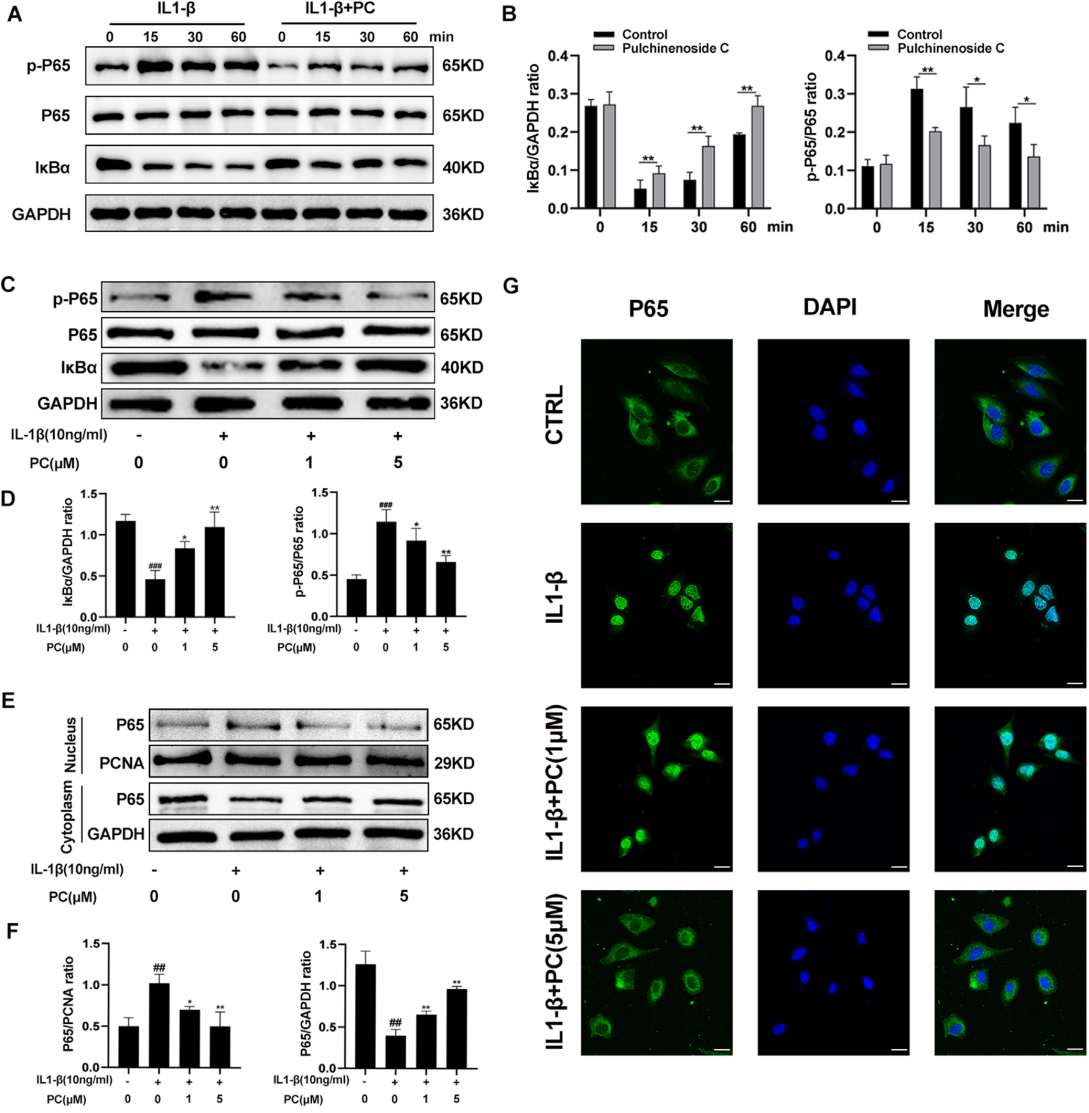
Effect of PC on NF‐κB signalling pathways in IL‐1β‐stimulated ADTC5 cells. (A, B) The expression levels of p‐P65 and IκBα at different time points after PC treatment were detected using western blotting and quantification analysis. (C, D) The relative expression levels of p‐P65 and IκBα after treatment with different concentrations of PC were detected using western blotting and quantitative analysis. (E, F) Expression level of p65 in the cytoplasm and nucleus and quantitative analysis. (G) The nuclear translocation of p65 was detected by immunofluorescence combined with DAPI staining of nuclei. Scale bar = 20 μm. The values presented are the mean ± SD of three independent experiments. ^##^ *p* < 0.01 and ^###^ *p* < 0.001 versus the control group and *p < 0.05, **p < 0.01 versus the IL‐1β alone treatment group.

**FIGURE 5:**
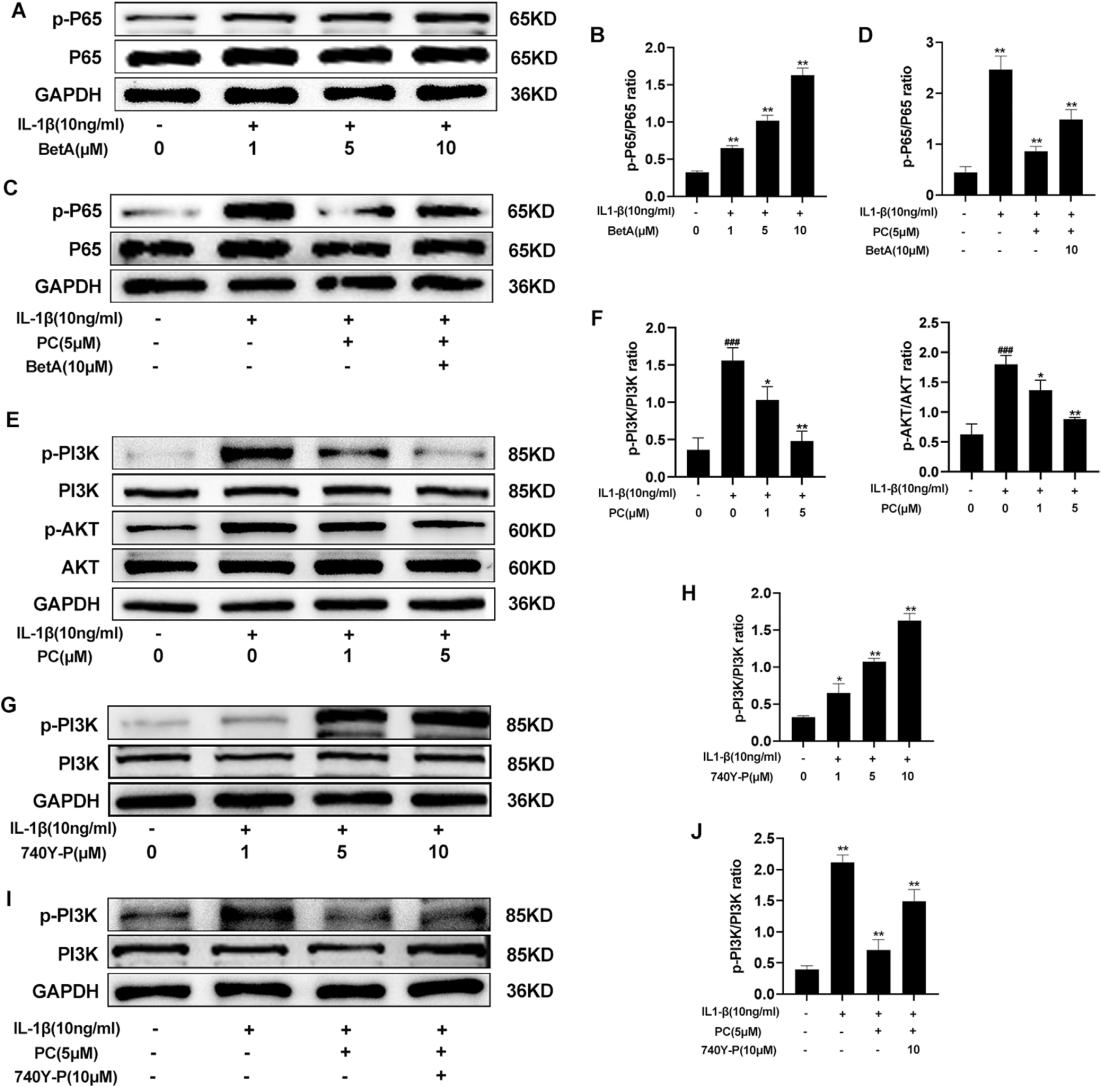
PC antagonised the activation of the PI3K/AKT/NF‐κB pathway. (A, B) BetA significantly increased the protein expression level of p‐P65 compared with that in the IL‐1β alone treatment group. (C, D) The relative expression levels of p‐P65 after treatment with PC and Bet A were detected using western blotting and quantitative analysis. (E, F) The relative expression levels of p‐PI3K and p‐AKT after treatment with different concentrations of PC were detected using western blotting and quantitative analysis. (G, H) 740Y‐P significantly increased the protein expression level of p‐PI3K compared with that in the IL‐1β alone treatment group. (I, J) The relative expression levels of p‐PI3K after treatment with PC and 740Y‐P were detected using western blotting and quantitative analysis. The values presented are the mean ± SD of three independent experiments. ^###^ *p* < 0.001 versus the control group and *p < 0.05 and **p < 0.01 versus the IL‐1β alone treatment group.

The transcription factor NF‐κB plays a key role in the occurrence and development of OA. Thus, NF‐κB inhibitors can be used to prevent joint damage in OA [26]. Western blotting was performed to detect the expression levels of IκBα and p65 in IL‐1β‐induced cartilage precursor ADTC5 cells to determine the effect of PC on the NF‐κB signalling pathway. IL‐1β stimulation significantly enhanced the degradation of IκBα and the phosphorylation of p65, indicating the activation of the NF‐κB signalling pathway in the cells. Pre‐treatment with PC significantly inhibited the activation of the NF‐κB signalling pathway (Figure 4A,B). Pre‐treatment of the cells with different concentrations of PC (1 and 5 μM) to further clarify the inhibitory effect of PC on the NF‐κB signalling pathway revealed that PC inhibited the phosphorylation of p65 and IκBα degradation in a dose‐dependent manner (Figure 4C,D). Proteins were extracted from the nucleus and cytoplasm to further clarify the effect of PC on the NF‐κB signal pathway. The results revealed that the p65 content in the nucleus increased significantly following treatment with IL1‐β; the addition of PC reversed this effect (Figure 4E,F). p65 immunofluorescence staining revealed that compared with that in the IL1‐β group, PC significantly blocked the nuclear translocation of p65 (Figure 4G). BetA (a new activator of NF‐κB) was added to the ADTC5 cells to verify the role of the NF‐κB signalling pathways in the mechanisms of PC on OA [27, 28]. Follow‐up experiments were conducted using 10 μM of BetA [29]. The addition of BetA significantly increased the phosphorylation level of p65 (Figure 5A,B). The optimal concentration of the agonist was selected to assess the relationship between PC and the NF‐κB pathway. Compared with that in the untreated cells, the NF‐κB pathway was activated when IL‐1β and NF‐κB agonists were added simultaneously. However, the activation of the NF‐κB pathway was inhibited upon the addition of PC (Figure 5C,D).

### 3.5 PC Inhibited PI3K/Akt Signalling

Activation of the PI3K/AKT signalling pathway plays a role in inducing synovitis, subchondral bone sclerosis, ECM destabilisation, chondrocyte apoptosis and autophagy in OA [12]. Analysis of the effect of PC on the activation of the PI3K/AKT signalling pathway revealed that IL‐1β activated the PI3K/AKT pathway, and PC (1 μM and 5 μM) inhibited the phosphorylation of PI3K and AKT induced by IL‐1β in a concentration‐dependent manner (Figure 5E,F). The PI3K activator 740Y‐P was used to activate the pathway to further investigate the effect of PC on the PI3K/AKT signalling pathway. An increase in the phosphorylation level of PI3K corresponding to the increase in the concentration of 740Y‐P was observed (Figure 5G,H). Subsequent experiments conducted using an appropriate concentration revealed that compared with that in untreated cells, the PI3K/AKT signalling pathway was significantly activated when IL‐1β and 740Y‐P were added. However, treatment with PC inhibited the activation of the PI3K/AKT pathway (Figure 5I,J). PC could reduce the inflammatory response by inhibiting the activation of the PI3K/AKT signalling pathway, thereby ameliorating OA.

### 3.6 PC Ameliorated OA Development in the DMM Mouse Model

**FIGURE 6:**
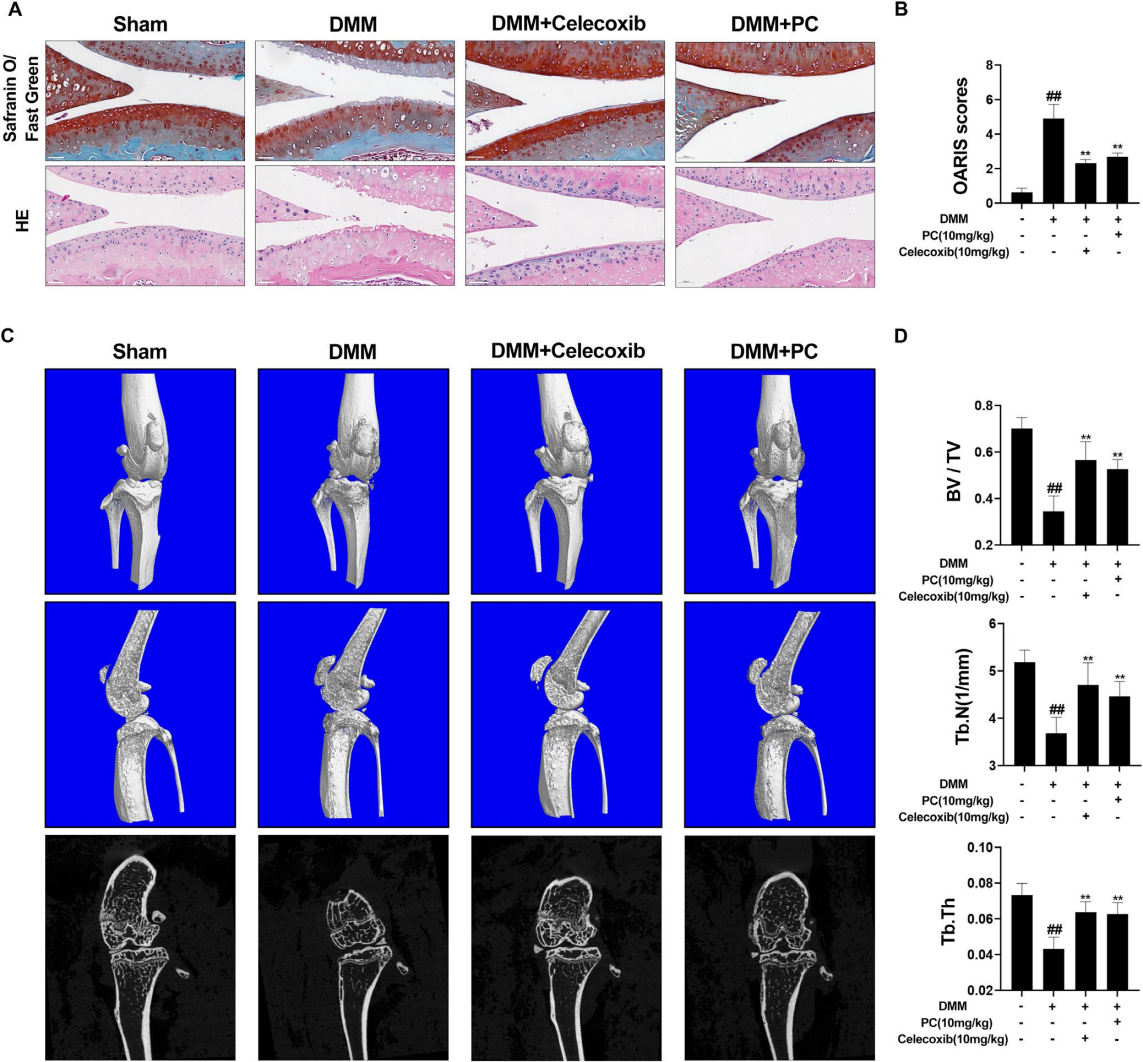
PC ameliorated OA progression in the DMM‐induced OA mouse model. (A) HE staining and Safranin O‐Fast Green staining showed the protective effect of PC or celecoxib on the knee joints of DMM‐induced mice. Scale bar = 50 μm. (B) OARSI scores showing cartilage structural damage in mice after DMM surgery. (C) Three‐dimensional micro‐CT imaging was performed to evaluate articular joint morphology in mice following treatment with PC or celecoxib. (D) Bone volume/tissue volume fraction (BV/TV), trabecular thickness (Tb.Th) and trabecular number (Tb.N). Differences between the groups are indicated as ^##^ *p* < 0.01 versus the sham group and **p < 0.01 versus the DMM group.

**FIGURE 7:**
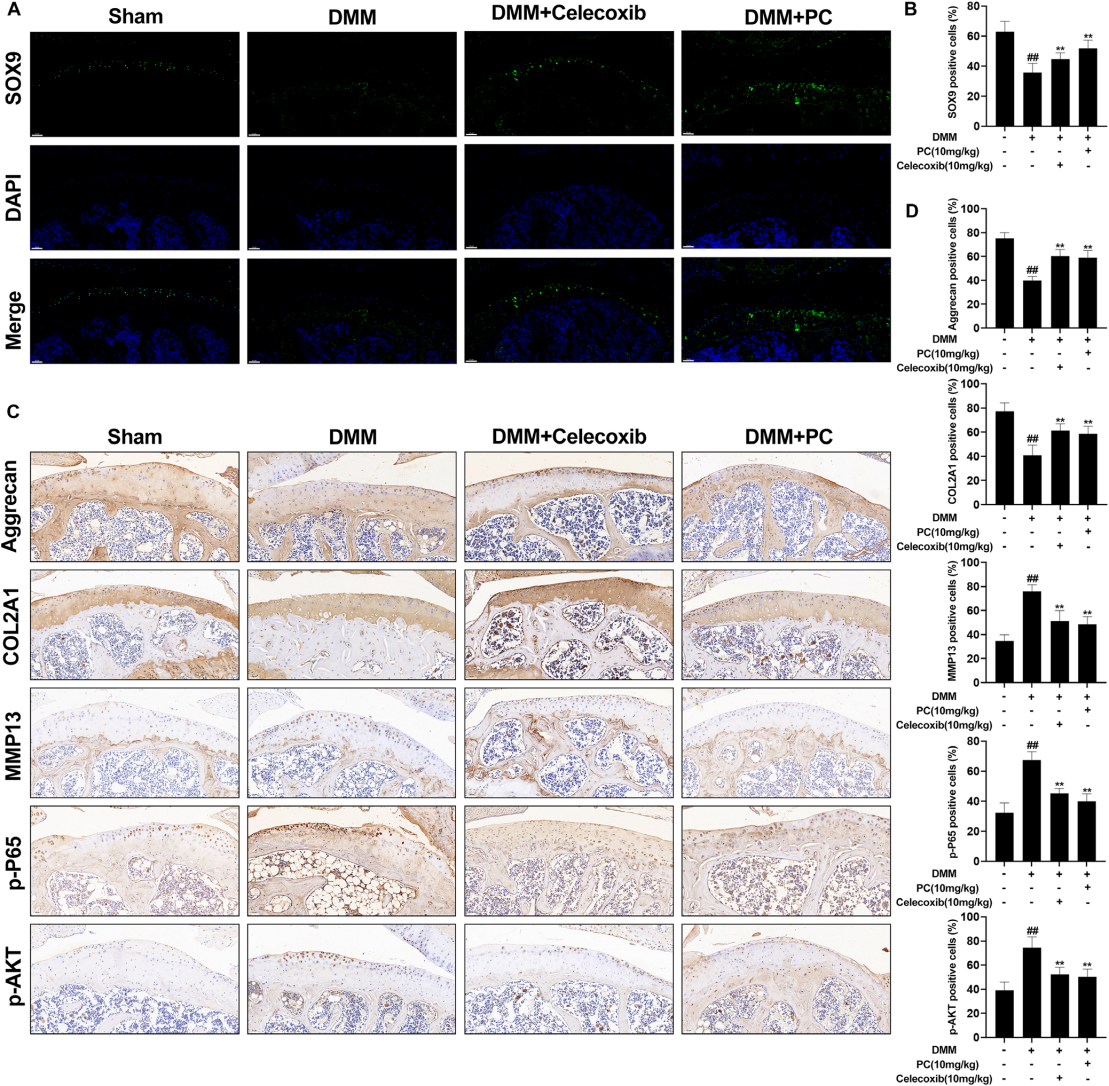
PC inhibits the PI3K/AKT/NF‐κB signalling pathway in a DMM‐induced OA mouse model. (A, B) Immunofluorescence of SOX9 in cartilage cells of the DMM‐induced OA model. Scale bars =50 μm. (C, D) Immunohistochemical staining (Aggrecan, Col2a1, MMP13, p‐P65 and p‐AKT) of mouse chondrocytes in articular cartilage and quantitative analysis. Scale bar = 50 μm. Differences between the groups are indicated as ^##^ *p* < 0.01 versus the sham group and **p < 0.01 versus the DMM group.

**FIGURE 8:**
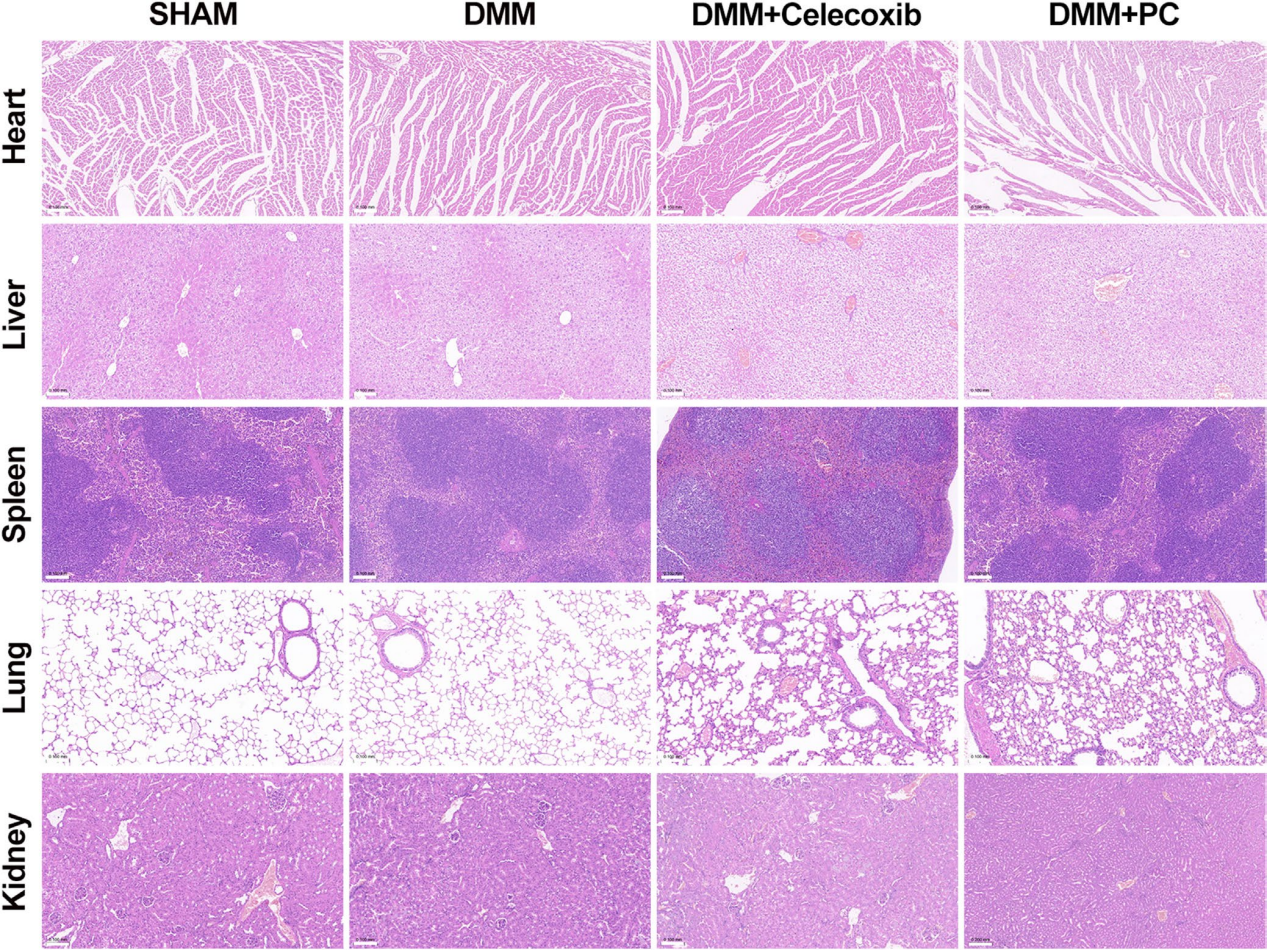
PC Analysis of in vivo toxicity of PC. HE staining of mouse heart, liver, kidney, lung and spleen. Scale bar = 100 μm.

A DMM model was established using C57BL/6 mice, and their knee joints were collected to validate the therapeutic efficacy of PC on OA. The knee joints were subjected to Haematoxylin and Eosin (H&E) staining, Safranin O‐Fast Green staining, and micro‐CT analysis. H&E and Safranin O/Fast Green staining revealed significant proteoglycan loss and cartilage erosion in the DMM group compared with that in the sham‐operated group. The cartilage surface was smoother in the PC‐treated group, and a notable increase in cell number and staining intensity was observed (Figure 6A). The OARSI scores were consistent with the staining results (Figure 6B). CT and three‐dimensional reconstructions of the mouse knee joints indicated an increase in osteophyte formation and subchondral bone sclerosis post‐DMM, particularly in the medial compartment, which exhibited severe subchondral bone damage. However, treatment with PC and celecoxib significantly mitigated the subchondral bone damage induced by DMM surgery. Analysis of BV/TV, Tb.Th and Tb.N revealed that treatment with PC reduced bone loss and preserved the structural integrity of the subchondral bone (Figure 6C,D). These findings indicate that PC effectively alleviates DMM‐induced subchondral bone degeneration, thereby delaying the progression of OA and reducing the gastrointestinal and cardiovascular side effects associated with the use of non‐steroidal anti‐inflammatory drugs (NSAIDs). Immunofluorescence staining was performed to investigate the protective effects of PC on the articular cartilage in mice. Consistent with the previous results, SOX9 gene expression was significantly reduced in the DMM group, whereas PC markedly inhibited the degradation of SOX9 induced by DMM surgery. The protective effect of PC on SOX9 appeared to be stronger than that of celecoxib (Figure 7A,B). The impact of PC pre‐treatment on the ECM was assessed using immunohistochemistry. Notably, the MMP13 levels in the DMM group were significantly higher than those in the sham group, whereas Col2a1 and aggrecan were markedly downregulated. Thus, PC inhibited the DMM‐induced expression of MMP13 and degradation of Col2a1 and aggrecan. Immunohistochemical staining for p‐AKT and p‐P65 was performed to elucidate the effect of PC on the PI3K/AKT/NF‐κB pathway in the DMM model. Phosphorylation of AKT and P65 was significantly increased in the DMM group compared with that in the sham group, whereas PC pre‐treatment reduced the number of p‐AKT and p‐P65 positive chondrocytes (Figure 7C,D). These findings indicate that PC may prevent the progression of OA in the DMM‐induced mouse model by blocking the PI3K/AKT/NF‐κB pathway. H&E staining performed to assess the effects of PC on the heart, liver, lungs, kidneys and spleen of the animals revealed no toxicity to the vital organs of the experimental mice (Figure 8).

### 3.7 Molecular Dynamics Simulation of PC Binding to P65 and AKT

PC mediates the development of OA by regulating the activation of the PI3K/AKT/NF‐κB signalling pathway. MD simulations of PC binding to AKT (1UNQ) and P65 (2O61) were performed to analyse the interaction between PC and receptor proteins. MD simulations of the protein‐ligand complexes were performed using Amber24 software, with the ff19SB force field and the OPC water model [30, 31, 32, 33].

**FIGURE 9:**
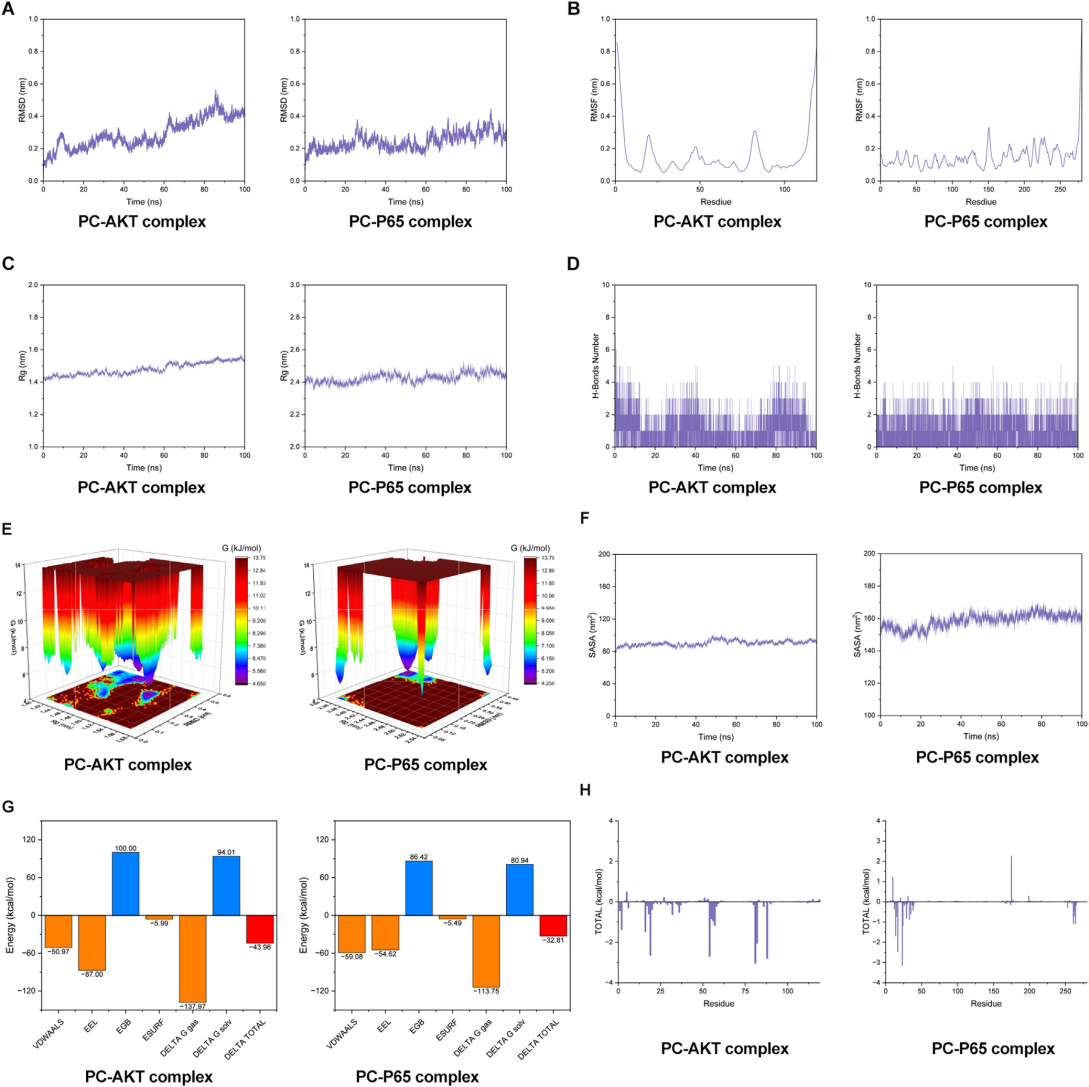
Molecular dynamics simulation of PC‐P65 and PC‐AKT complexes. (A) The root mean square deviation (RMSD) curves of the PC‐P65 and PC‐AKT complexes. (B) The root mean square fluctuation (RMSF) curves of the PC‐P65 and PC‐AKT complexes. (C) The radius of gyration (Rg) curves of the PC‐P65 and PC‐AKT complexes. (D) The H‐Bonds number of the PC‐P65 and PC‐AKT complexes. (E) The free energy landscape (FEL) maps of the PC‐P65 and PC‐AKT complexes. (F) The solvent‐accessible surface area (SASA) of the PC‐P65 and PC‐AKT complexes. (G) The average binding free energies of the PC‐P65 and PC‐AKT complexes. (H) The energy contribution of amino acid residues in the PC‐P65 and PC‐AKT complexes.

The RMSD curves of the PC‐P65 and PC‐AKT complexes were validated. The RMSD fluctuations were maintained within 0.2 nm and stabilised after a certain simulation time, indicating that both systems maintained stable structures (Figure 9A). RMSF analysis was performed to evaluate the fluctuations of amino acid residues during the simulations. The RMSF curves for amino acids in the 1UNQ and 2O61 complexes predominantly fluctuated within 1 nm. No significant deviations were observed except for terminal residues, which exhibited reasonable fluctuations of close to 1 nm. This finding suggests that the incorporation of the small molecule had minimal impact on the stability of amino acid residues and that the complexes remained stable (Figure 9B). The radius of gyration (Rg) was used to assess the compactness and stability of the structures, with larger Rg values indicating significant expansion of the system during MD simulations. The Rg fluctuations of the PC‐AKT and PC‐P65 complexes were maintained within 2.5 nm and stabilised over time, indicating no significant variations. This finding suggests that PC formed stable and compact complexes with AKT and P65 without inducing major structural changes in the proteins. The Rg of the PC‐AKT complex was slightly smaller than that of the PC‐P65 complex. This finding indicates that PC forms a more stable complex with AKT (Figure 9C). The number of hydrogen bonds contributing to the stability of the PC‐protein complexes was calculated to evaluate the hydrogen bonding interactions at the binding sites. The number of hydrogen bonds between PC and AKT was > 3 throughout the simulation, with a maximum of eight hydrogen bonds being observed (Figure 9D). This finding indicates a strong interaction and high complex stability. The PC‐P65 complex maintained at least three hydrogen bonds, with a maximum of five bonds being observed, suggesting good stability. PC exhibited a stronger and more stable interaction with AKT. Free energy landscape (FEL) maps were generated to describe the most stable conformations during MD simulations. The dark purple/blue spots in Figure 9E indicate the lowest energy states, representing the most stable structures, whereas the red/yellow spots correspond to unstable conformations. The PC‐AKT complex predominantly occupied conformations with RMSD values of 0.3–0.4 nm and Rg values of 1.50–1.54 nm or RMSD values of 0.45–0.55 nm and Rg values of 1.44–1.48 nm, indicating greater structural stability. The PC‐P65 complex was most stable in the RMSD range of 0.35–0.45 nm and Rg range of 2.35–2.38 nm or RMSD range of 0.05–0.10 nm and Rg range of 2.35–2.37 nm. Solvent‐accessible surface area (SASA) is another key factor that can be used to assess protein folding and stability, as proteins with stable structures tend to have lower SASA values. The SASA curve of the PC‐AKT complex remained nearly unchanged throughout the simulation, indicating its structural stability. Similarly, the SASA curve of the PC‐P65 complex decreased gradually and then stabilised, further confirming its stability. The SASA values of the PC‐AKT complex were lower than those of the PC‐P65 complex, indicating greater stability (Figure 9F). The binding free energy for both systems was calculated after confirming the stability of the complexes. The average binding free energies of the PC‐AKT and PC‐P65 complexes were −43.96 kcal/mol and −32.81 kcal/mol, respectively, indicating strong interactions. The binding affinity of PC for AKT was stronger than that of P65 (Figure 9G). The contributions of key amino acid residues at the binding interfaces were also analysed. Residues GLN 81, ARG 88, LEU 54, GLU 19, TRP 82, LYS 16, SER 2, PHE 57, ASN 56 and ASN 55 were involved in binding in the PC‐AKT complex. Residues ARG 23, ARG 17, MET 14, SER 24, GLU 264, GLU 261, SER 33, PRO 29, ARG 15 and LEU 262 were involved in binding in the PC‐P65 complex (Figure 9H).

**FIGURE 10:**
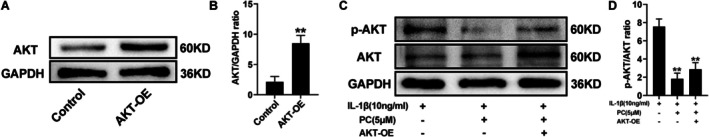
The molecular interaction between PC and AKT leads to significant suppression of AKT hyperphosphorylation. (A, B) The expression level of AKT following transfection with the AKT overexpression plasmid was detected by Western blotting and quantitative analysis. (C, D) Western blotting and quantitative analysis were used to detect the relative expression level of p‐AKT after PC or AKT1 overexpression lentivirus treatment. **p < 0.01 versus the control group.

These findings suggest that PC interacts with AKT and P65 to inhibit the activation of the PI3K/AKT/NF‐κB signalling pathway. MD simulations revealed that PC exhibited a more stable binding affinity for AKT. Therefore, an AKT overexpression plasmid was constructed to validate these results in vitro. A significant increase in the AKT expression levels was observed following transfection with the AKT overexpression plasmid (Figure 10A,B). Furthermore, in vitro experiments revealed that PC inhibited AKT phosphorylation, as the phosphorylation level of AKT decreased compared with that in the control group upon treatment with PC (Figure 10C,D).

## 4 Discussion

Osteoarthritis (OA), a critical chronic whole joint disorder affecting older individuals that causes severe impairment, poses a substantial financial burden on society [34]. NSAIDs and glucosamine yield only a temporary reduction in the discomfort caused by OA; moreover, long‐term use may be associated with serious side effects [35, 36]. Therefore, innovative therapies that can delay the progression of OA and improve clinical prognosis must be developed. Chinese herbal medicine extracts have attracted attention owing to their direct origin from herbs, high safety and fewer side effects. Traditional Chinese medicine can relieve cartilage inflammation and adjust the metabolic balance of subchondral bone in patients with OA, thereby providing a more moderate and long‐term solution [37].

The natural pulsatilla saponin B4 (PC, also known as Anemoside B4) possesses potent antitumour, neuroprotective and anti‐inflammatory properties. PC exerts a definite anti‐inflammatory effect in the treatment of acute liver injury and pneumonia caused by *Klebsiella pneumoniae* and influenza virus FM1; however, its effectiveness in chronic OA remains unknown [38, 39]. The present study investigated the effect of PC on the inflammatory response of chondrocytes in vivo and in vitro [40].

OA is a common degenerative disease characterised by the destruction of joint cartilage and chronic inflammation of peripheral tissues resulting from stimulation by multiple inflammatory factors. MMPs, particularly MMP13, play a role in cartilage deterioration through their unique capacity to break down type II collagen [41]. Overexpression of ADAMTS5 stimulates the degradation of aggrecan, a major structural proteoglycan in cartilage, when inflammatory stimuli are present [42]. Previous studies have explored the utility of treating OA with medications that prevent the synthesis of ADAMTS and MMPs. SOX9 expression is significantly reduced in osteoarthritic articular cartilage. SOX9, a cartilage formation regulator, controls numerous downstream components involved in the sequential events of chondrogenesis in a phased manner to enhance the expression of COL2a1 and aggrecan [43]. Inducible nitric oxide synthase (iNOS) is expressed when inflammatory cytokines, such as interleukin, are exposed to arthritic cartilage. This results in the excessive release of NO, which inhibits the production of collagen and proteoglycans and promotes the expression of MMPs [44]. In addition, COX‐2, which mediates the considerable production of MMPs and increases the progression of OA, is triggered by inflammation [45]. The findings of the present study demonstrated that PC prevents the secretion of inflammatory factors (TNF and IL6) in response to IL‐1. PC reduced the expression of COX‐2 and iNOS in a dose‐dependent manner. PC dramatically slowed the inflammation‐induced production of MMP13 and ADAMTS5, as well as the degradation of type II collagen and SOX9 in ADTC5 cells.

The pathogenesis of OA remains elusive. However, several cellular pathways have been implicated in the development and progression of OA. NF‐κB comprises an isotype and heterodimer of five Rel family members, namely, NF‐κB 1 (P105/P50), NF‐κB 2 (P100/P52), RelA (p65), RelB and C‐Rel. The P50/65 heterodimer is the most abundant Rel dimer in cells. NF‐κB dimers are present in the cytoplasm as inactive forms after binding to the IκB protein [46, 47]. Following stimulation by IL1‐β, IκB is phosphorylated by IκB kinases (IKKs) and degraded by the proteasome. The free NF‐κB complex is transported to the nucleus, which activates the expression of proinflammatory and destructive mediators (e.g., cyclooxygenase 2 [COX2] and prostaglandin E2 [PGE2], inducible nitric oxide synthase [iNOS] and catabolic enzymes [ADAMTS5 and MMP13]) [48, 49, 50]. The present study demonstrated that PC could inhibit the translocation of P65 to the nucleus and the degradation of IκBα, reduce the expression of inflammatory factors and inhibit the secretion of MMPs and ADAMTS proteins, thereby delaying the progression of OA.

**FIGURE 11:**
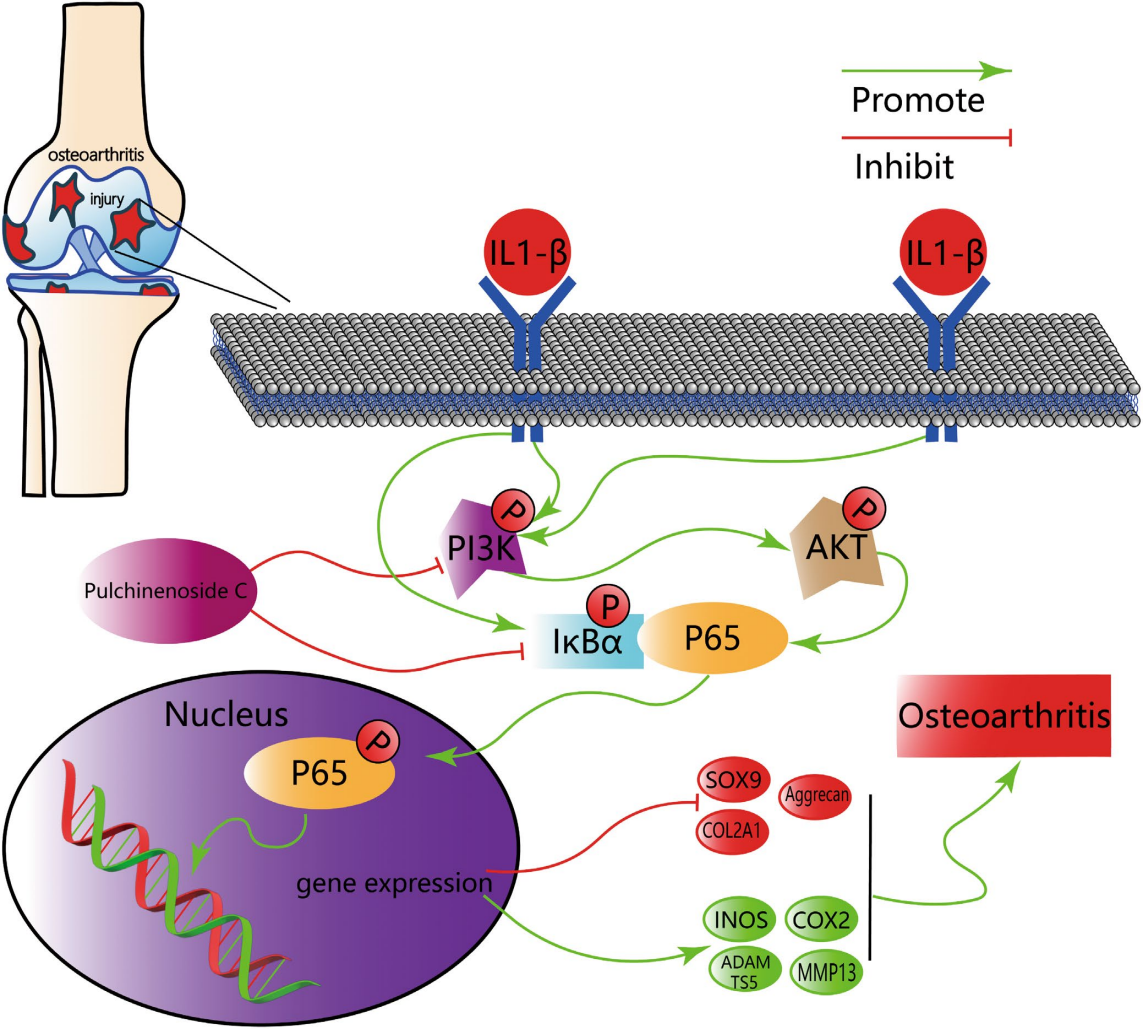
Schematic diagram illustrating the role of PC in cartilage degeneration and OA progression. Schematic diagram of the potential protective effects of PC on OA progression. The red arrow indicates inhibition. The green arrow indicates the promotion effect.

The present study also explored the effect of PC on the PI3K/AKT signalling pathway. The PI3K/AKT signalling pathway is a complex signalling pathway with various regulatory and effector factors that mediate apoptosis in chondrocytes and participate in the metabolism of subchondral bone in arthritis [51, 52]. PI3K and AKT can be rapidly phosphorylated following IL1‐β stimulation [16]. Inhibiting the phosphorylation of PI3K/AKT and NF‐κB can significantly reduce the occurrence and development of inflammation [53]. PI3K/PKA (protein kinase A)/AKT can mediate the degradation of IκBα and the phosphorylation of p65/RelA by affecting IκB kinase (IKK), resulting in the activation of the NF‐κB inflammatory pathway and the increased production of MMPs and ADAMTS [54]. However, treatment with PC results in a dramatic decrease in the p‐PI3K and p‐AKT levels, indicating that PC inhibited their activation. PI3K/AKT may be involved in IL‐1β‐induced activation of NF‐κB. PC reduced IL‐1β‐induced cartilage inflammation by inhibiting the activation of the PI3K/AKT/NF‐κB pathway. Figure 11 illustrates the underlying mechanism.

A mouse model of DMM‐induced OA was constructed to further verify the effect of PC on OA. Micro‐CT scanning and analysis of the mouse knee joint revealed that PC can alleviate the subchondral bone destruction caused by DMM surgery and that its therapeutic effect is comparable with that of NSAIDs. HE staining and Safranin O‐Fast Green staining revealed that the joints of the PC‐treated mice, with smoother articular surfaces and a greater abundance of matrix and associated chondrocytes, resembled those of the sham‐operated mice more closely than those of the mice in the DMM group. Col2a1 and aggrecan are the main components of ECM, and their destruction plays a crucial role in the onset and progression of early arthritis. MMP13 is a pivotal inflammatory factor that mediates this effect. The present study demonstrated that PC can delay the degradation of cartilage ECM in arthritic mice and maintain the relative homeostasis of articular cartilage. This may be attributed to the inhibition of the PI3K/AKT/NF‐κB signalling pathway by PC.

Despite the encouraging in vitro and in vivo results, the present study has some limitations. First, PC is administered via intraperitoneal injection, rather than orally or via intra‐articular injection. Second, only the inhibitory effect of PC on chondrocyte inflammation through its effect on the activation of the PI3K/AKT cell pathway and the NF‐κB cell signal pathway was confirmed herein. The direct relationship between them could not be proven. Lastly, only the PI3K/AKT/NF‐κB pathway was selected to demonstrate the effect of PC on OA owing to the complex occurrence and development mechanisms of OA.

In summary, the present study systematically revealed the dual regulatory mechanisms of natural pulsatilla saponin B4 (PC) in OA treatment through in vitro and in vivo experiments. PC effectively suppressed the release of IL‐1β‐induced pro‐inflammatory cytokine and ECM degradation in chondrocytes in vitro. In vivo, it significantly delayed the progression of early arthritis in mice with DMM‐induced OA by targeting the PI3K/AKT/NF‐κB signalling pathway. MD experiments further validated the interactions between PC and key signalling nodes, providing a theoretical basis for its multi‐target intervention properties. The administration route warrants optimisation; nevertheless, PC, a natural herbal‐derived active compound, possesses the dual advantages of potent anti‐inflammatory and cartilage‐protective effects, offering a novel avenue for the development of safe and long‐acting OA therapeutic strategies. Future studies must aim to explore the clinical translation potential of PC and further elucidate its molecular networks in reconstructing joint homeostasis by modulating the chondro‐osseous metabolic microenvironment, thereby addressing the urgent requirement for developing OA prevention and treatment strategies in the context of an aging society.

## Author Contributions

**Jiawei Hu:** conceptualization (lead), formal analysis (lead), writing – original draft (lead), writing – review and editing (equal). **Kai Xiao:** software (equal), writing – review and editing (equal). **Jianhui Liang:** methodology (lead), writing – review and editing (equal). **Xiaolong Yu:** writing – original draft (supporting). **Meisong Zhu:** investigation (equal), resources (supporting). **Zhihui Kuang:** formal analysis (equal). **Shoujie Shi:** investigation (equal). **Bin Zhang:** funding acquisition (equal). **Qiang Xu:** funding acquisition (equal), writing – review and editing (equal).

## Ethics Statement

All experiments were approved by the Laboratory Animal Science Center of Nanchang University. All animal experiments and surgical procedures were performed according to the guidelines of the Animal Care Committee of Nanchang University (Ethical Approval Number: DM20210410).

## Conflicts of Interest

The authors declare no conflicts of interest.

## Data Availability

Data will be made available on request.
